# Supplementary material for: Chronic Exposure to the Food Additive tBHQ Modulates Expression of Genes Related to SARS-CoV-2 and Influenza Viruses
Source: Life (Basel). 2022 Apr 26;12(5):642. doi: 10.3390/life12050642 (PMC9147452; doi:10.3390/life12050642)
Supplement: Supplementary file 1 [file life-12-00642-s001.zip › life-1689160-supplementary.pdf]

Supplementary Figure S1. Change in body weight over treatment time. Mice were maintained on food pellets supplemented with 1.5% (w/w) tBHQ or vehicle for 20 days. Weight of animals is expressed as percentage of original body weight measured at the first day of the treatment period. Data are means ± SD of 6-6 mice/group

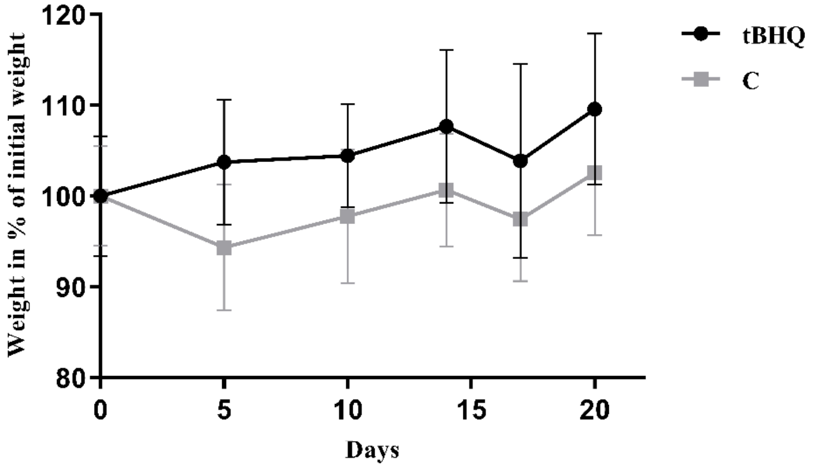

Supplementary Figure S2. Representative scatter plot of $CD3^+$ positivity of $CD4^+$ cells. $CD4^+$ cells were isolated from splenocytes derived from tBHQ fed and control mice with magnetic separation. CD3 positivity of these cells were determinated with FACS analysis.

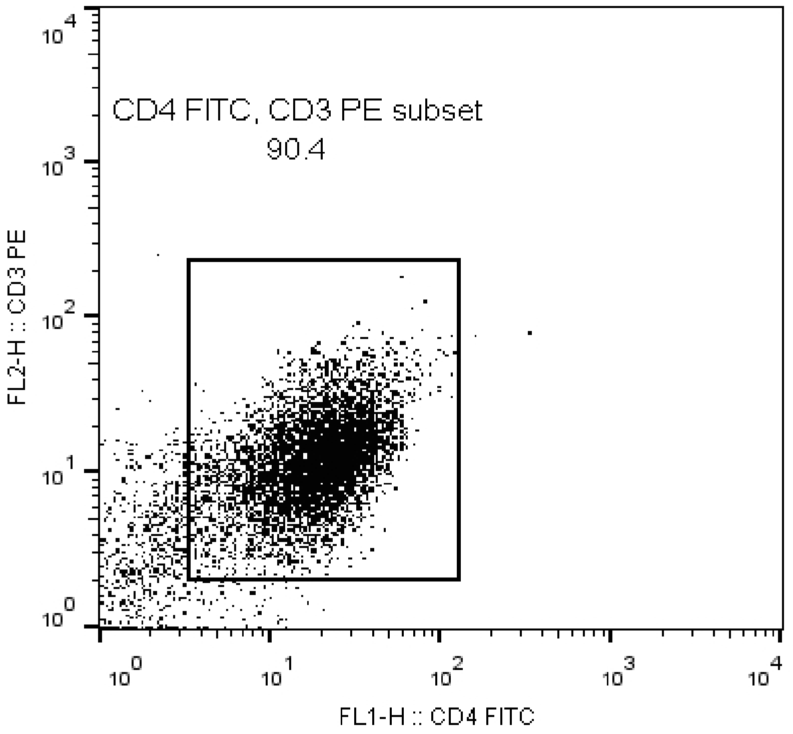

Supplemetary Figure S3. Correlation between cDNA microarray and RT-PCR data. The fold changes in transcript levels of 47 selected genes determined by both methods were log2 transformed, and the values were plotted against each other to evaluate their correlation.

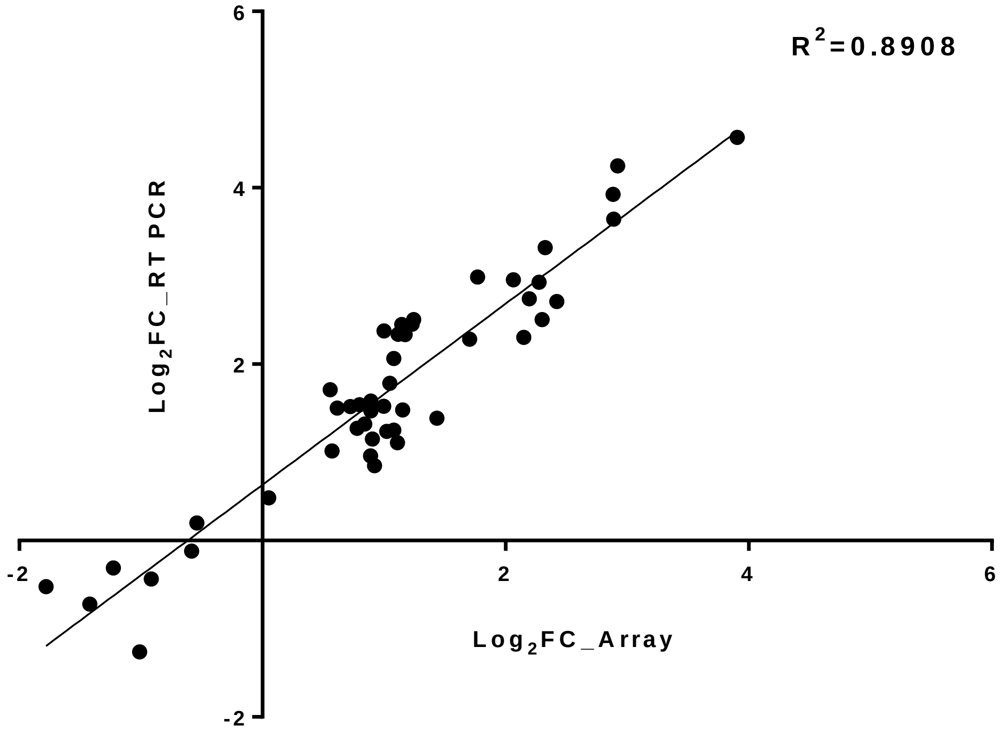

Supplementary Table S1. List of significantly changed genes after per os tBHQ treatment measured with Agilent microarray. In total 269 genes were found with a significantly changed expression in tBHQ treated, splenic $CD4^+$ cells compared to control ($_{nom}p<0.05$). From these, altogether 47 genes were validated back by RT-PCR (marked with bold asterisk [*****])

Up-regulated genes			Down-regulated genes		
**GeneName**	**logFC**	**adj.P.Val**	**GeneName**	**logFC**	**adj.P.Val**
Stfa1*	3.9064895	0.0169437	*Ubn1*	-0.391629	0.0497032
LOC433016	3.7924452	0.0169437	*Cenpl*	-0.400142	0.0490856
Stfa2l1	3.5874328	0.0169437	*Chic2*	-0.426049	0.0429823
ENSMUST00000077110	3.4803365	0.0162617	*Serbp1*	-0.429634	0.0462228
2300002D11Rik	3.3350887	0.0409651	*Cenpa*	-0.446086	0.0408644
Hp	3.0378437	0.0208273	*Amd1*	-0.482322	0.0415054
Stfa2*	2.9227256	0.0169437	*Camk2a*	-0.485021	0.0445632
Retnlg	2.9129956	0.02052	*0610040D20Rik*	-0.485044	0.0391519
Il8rb	2.8891376	0.0107003	*Rybp*	-0.504705	0.0385746
Mmp8*	2.8853301	0.0169437	*Sh3rf1*	-0.504826	0.0499011
Cxcl2	2.8182754	0.0379543	*Usp38*	-0.506739	0.0348195
Clec4e	2.7445173	0.0324984	*Lemd3*	-0.50696	0.0430313
Il8rb	2.6500234	0.0162617	*Siah2*	-0.508708	0.0480091
Clec4d	2.5828577	0.0310919	*Dnajb6*	-0.517009	0.0244566
Chi3l1	2.4505219	0.02052	*Prickle1*	-0.518386	0.0395634
S100a8*	2.4212595	0.0107003	*3230401D17Rik*	-0.518576	0.0265968
G0s2	2.3701683	0.0199841	*Bcl10*	-0.518704	0.0310919
s100a9*	2.3268041	0.0169437	*AK013994*	-0.526674	0.0461562
Ppp1r3d	2.3008787	0.0204766	***Tiprl****	-0.539461	0.0408163
Lcn2*	2.2997459	0.0204857	*Pde4b*	-0.541338	0.0435927
Trem3*	2.27623	0.0169437	*Peg12*	-0.545333	0.0482127
Chi3l3	2.2595454	0.0491891	*1810009J06Rik*	-0.545503	0.0406771
Csf3r	2.2523436	0.0208386	*Srfbp1*	-0.546509	0.0435377
Mrgpra2	2.2362744	0.0169437	*Dnajb6*	-0.548789	0.0337991
Mmp9*	2.1942358	0.0107003	*Rkhd3*	-0.558443	0.0379543
Gad1*	2.1500906	0.0169437	*Tgif2*	-0.565342	0.046275
Arg2	2.0986002	0.0107003	*1700054N08Rik*	-0.573559	0.0234741
Trem1*	2.0639241	0.0236857	*Klra3*	-0.577878	0.0360263
1100001G20Rik	2.0575514	0.0216319	***P2ry14****	-0.582939	0.035137
Hdc	1.8824383	0.037632	*Sox4*	-0.583874	0.0411697

Nfe2	1.8673312	0.008929	*NAP007437-001*	-0.584212	0.0360263
Emilin2	1.8174195	0.0204766	*Gem*	-0.587821	0.0424369
BC089618	1.8101363	0.026206	*Rngtt*	-0.589558	0.0370012
Alox5*	1.7709131	0.0204766	*Dapp1*	-0.590742	0.0264522
Lrg1	1.7523144	0.0304426	*Slc22a2*	-0.596808	0.0497032
Ccl6*	1.703382	0.0179544	*Ptprf*	-0.597726	0.0264522
Lilrb4	1.6770058	0.0149756	*Kbtbd2*	-0.600365	0.0332443
Dfna5h	1.6426888	0.02052	*Glt28d2*	-0.601611	0.0480091
Mtus1	1.5887229	0.0216319	*Hbs1l*	-0.604691	0.0264522
Cebpd	1.5652603	0.0204857	*C030040K24Rik*	-0.612019	0.0424333
Chi3l4	1.4962512	0.0373994	*Tob2*	-0.62171	0.0310919
Fndc1	1.4674064	0.0199841	*Grb7*	-0.637942	0.040137
Alox5*	1.4654162	0.0317135	*Bhlhb2*	-0.638606	0.0208386
Il1rn*	1.4345521	0.0230512	*Myb*	-0.649523	0.0215293
Padi4	1.4258104	0.0424369	*Hmgn3*	-0.662442	0.0480091
Mt1	1.4242178	0.0469002	*Ptprf*	-0.671418	0.0289639
Fpr-rs2	1.4077917	0.0199841	*Atf3*	-0.678332	0.0310919
Pi16	1.4067729	0.03911	*Otud1*	-0.680716	0.0217045
Emilin2	1.4062323	0.0241828	*Ift80*	-0.691938	0.0356522
2300003P22Rik	1.402857	0.0208273	*Skil*	-0.692136	0.046919
Mtus1	1.4020095	0.0230512	*Riok1*	-0.694497	0.040137
Tnfaip2	1.3847628	0.0359041	*Tieg3*	-0.695883	0.0445632
Klra2	1.3470671	0.0264522	*Pik3r3*	-0.707284	0.0482127
Fpr1	1.2951568	0.0351075	*Ube1l2*	-0.71087	0.0406771
Ms4a8a	1.2753775	0.0292462	*Jmy*	-0.71449	0.0469002
Siglece	1.265304	0.008929	*BC057022*	-0.729287	0.0208386
Mrap	1.2580866	0.0199841	*Dyrk3*	-0.738454	0.0241828
Gcnt2	1.2471069	0.0365111	*Hpse*	-0.743195	0.0334709
A530064D06Rik	1.2435046	0.0179544	*9630054F20Rik*	-0.746661	0.0199841
Ifitm1*	1.2428823	0.0216319	*D230015J17Rik*	-0.75302	0.0395634
Edn1	1.2405578	0.0359041	*Efnb2*	-0.75834	0.0241828
5730557B15Rik	1.2404816	0.0424369	*Tnfsf11*	-0.76561	0.0434025
Nup155	1.2339759	0.0204857	*Zfyve28*	-0.772459	0.0208386
Cd33*	1.2316716	0.0365111	*Lrrc16*	-0.779191	0.0346277
Mboat2	1.2297455	0.0208386	*5730596K20Rik*	-0.781622	0.0236857
Sfxn5	1.2135636	0.0415054	*Mogat1*	-0.782331	0.0216319
Gzmb	1.2117054	0.0422067	*Fosl2*	-0.790418	0.0264522
Ms4a6d	1.1988909	0.0334709	*Frat2*	-0.805562	0.0179544
Slfn3	1.181386	0.0389148	*Foxj3*	-0.823862	0.0323347
2810417M05Rik	1.1796615	0.0264522	*Crem*	-0.85436	0.0463652
Tgfbi	1.1743436	0.0169437	*Rel*	-0.85632	0.0480807
Ifitm2*	1.1740416	0.0179544	*Tnfrsf17*	-0.861409	0.0264522
BC055004	1.1692564	0.0466345	*Zfand2a*	-0.863498	0.0261117
Slc16a3	1.1640191	0.0241828	*2310050B05Rik*	-0.899224	0.0445632
Padi4	1.1560191	0.0409659	*Plk2*	-0.904059	0.0304426

Cd14*	1.1540813	0.0179544	***Dusp4****	-0.913381	0.0354384
AK087372	1.151899	0.0329407	***Nfil3****	-0.914336	0.0409659
Mmp7*	1.146066	0.0216319	*Tnfsf4*	-0.915992	0.0265968
Stac2	1.1384134	0.0236221	*Slco4a1*	-0.925644	0.0318903
Ms4a11	1.1302852	0.0304426	***Dusp4****	-0.952144	0.0169437
Drd3*	1.1149364	0.0360263	*Tmem163*	-0.958325	0.0387303
LOC384622	1.110668	0.0199841	*Snf1lk*	-0.979634	0.0169437
Apoe*	1.1101803	0.0179544	*Nfil3*	-1.010053	0.02052
0610040J01Rik	1.109578	0.0379543	*Hey1*	-1.028181	0.0495035
Gnaz	1.1011279	0.0289639	*9830130M13Rik*	-1.149571	0.0478532
Pla2g7*	1.0815618	0.0268795	*Slc41a2*	-1.210258	0.0199841
Ccrl2*	1.0814998	0.0359041	*Hs3st1*	-1.218633	0.0424369
Myo18b	1.0793971	0.0485988	***Dusp14****	-1.227388	0.0230512
Slc11a1	1.072413	0.0208273	*Klk1b4*	-1.237813	0.0463652
2310005C01Rik	1.0722742	0.0337991	*Zc3h12c*	-1.256982	0.0199841
C1qa	1.062227	0.0199841	*Mgl1*	-1.411163	0.0208273
3230401I01Rik	1.0601258	0.0200108	***Dusp10****	-1.419826	0.0107003
Syn2	1.0577219	0.0334709	*Gzma*	-1.424467	0.0264522
Hs3st2	1.0513419	0.0310919	*Epha2*	-1.444171	0.0199841
Il1b*	1.0480056	0.0482127	*2210020M01Rik*	-1.516391	0.0264522
Lrp1	1.0403633	0.0215201	*Klk1b5*	-1.585054	0.0433009
Ier3	1.0387872	0.0310919	*Upb1*	-1.59126	0.0241828
AI452102	1.0372	0.0264522	*Siglech*	-1.695752	0.0409659
9830107B12Rik	1.0362593	0.0318903	***Cd209d****	-1.78035	0.0230512
C1qb	1.0350895	0.0346277	*Cox6a2*	-1.809345	0.033583
Clec7a	1.0304137	0.0409651			
Ifitm2	1.0288298	0.026206			
C6	1.0250447	0.0406771			
Gas6	1.0244309	0.0290369			
Mgst1*	1.0233708	0.0230512			
Sepn1	1.023357	0.0208386			
LOC676870	1.0232467	0.0356522			
6030422H21Rik	1.0144226	0.0208273			
Pira3	1.0008394	0.0208386			
Adamts20	0.9989663	0.0424369			
Pira6*	0.9986171	0.0497032			
C130076O07Rik	0.9926432	0.0179544			
Centd3	0.9923345	0.0406771			
Mgst1	0.9779364	0.0408644			
Pdgfc	0.9761474	0.0489969			
Cyp4f18	0.9730086	0.0204766			
Pira3	0.96965	0.0204857			
Tspan4	0.9643841	0.0200108			
Pax6	0.9584796	0.032949			
Sult1a1	0.9559812	0.0334709			

Pygl	0.9442645	0.0406771			
Lgmn	0.9439797	0.0265208			
Lhfp	0.9433504	0.0354384			
Pilra	0.9322316	0.0395634			
Rab3il1	0.9315289	0.0169437			
Kcnj16	0.927932	0.0334709			
2310076L09Rik	0.9270255	0.0318903			
Il13ra1*	0.9225341	0.0360263			
Sepx1	0.9184596	0.0445632			
Fcgr3	0.9175912	0.0365111			
Mt1	0.9158274	0.0228018			
Cav1*	0.9043254	0.0369571			
Ptgs2*	0.8921205	0.0265208			
P2ry2*	0.8916829	0.0264522			
C130050O18Rik	0.8906791	0.0264522			
Nr1h3*	0.8889046	0.0264522			
Tnfsf13	0.8804367	0.0462228			
1200002N14Rik	0.8796729	0.0199841			
LOC381484	0.8791967	0.0354384			
Ptplad2	0.8612147	0.0256834			
Fut7	0.861012	0.0379543			
Lypla3	0.8600306	0.0462228			
Hfe	0.8493904	0.026206			
Myo1d	0.8474245	0.0365111			
Foxo3a	0.8466951	0.0433009			
Ptgs1	0.8414499	0.0370951			
Tirap*	0.8337591	0.0379543			
Csf1r	0.832827	0.0429823			
Hck	0.8316354	0.0179544			
Csf1r	0.8272789	0.0310919			
Slc45a3	0.8199816	0.0204766			
Vcam1	0.8125966	0.0395634			
8030453O22Rik	0.8102447	0.0409651			
Anxa2	0.8023203	0.0169437			
Ccl24*	0.7978902	0.0334709			
Clec4a2	0.7969552	0.0304426			
Scamp5	0.7961883	0.0199841			
Csf1r	0.7938383	0.0354384			
Plxnb2	0.7936975	0.0236857			
Cab39l	0.7883911	0.0208386			
Jundm2	0.7850421	0.0169437			
Lyzs	0.7836424	0.0304426			
Tirap	0.7775237	0.0208386			
Hk3	0.7731507	0.0184191			
ENSMUST00000039068	0.7708831	0.037219			

Gpd1	0.7644566	0.0354384			
Gcnt1	0.756508	0.026206			
Ccl24	0.7499391	0.0334709			
Osm	0.7480422	0.02052			
Tcn2	0.7441501	0.0379543			
Amhr2	0.7428653	0.0480807			
Pdgfc	0.7380453	0.0469002			
Aldh3b1	0.737373	0.0409651			
Rcsd1	0.7366419	0.0241828			
Gpr141	0.7305162	0.0169437			
Gcnt1	0.7297506	0.0302708			
Dok3	0.7248771	0.0208386			
Camk1*	0.7239797	0.0409659			
Mertk	0.7226912	0.0435927			
AK036708	0.7214743	0.0408644			
Cr2	0.7188317	0.0430313			
B430306N03Rik	0.709819	0.0377836			
Anxa2	0.7078039	0.0199841			
Scd2	0.7054011	0.0395634			
Ppap2b	0.7043332	0.0424333			
Lgals3	0.7022757	0.0395634			
6430548M08Rik	0.7003627	0.03911			
Hip1	0.6990555	0.026206			
AK041774	0.6948503	0.0409659			
Prf1	0.6925697	0.0387303			
Lilrb3	0.6915752	0.0358353			
Ela1	0.6904486	0.0343527			
Mapk13	0.6838903	0.0317135			
Stard8	0.6836808	0.0370012			
Ncf2	0.6823333	0.0439583			
Ninj1	0.6747365	0.0179544			
Galc	0.6710196	0.0217501			
Popdc2	0.6696056	0.0260572			
Fcer1g	0.6690582	0.0313892			
Nupr1	0.6630438	0.0478532			
Fcer1g	0.6598249	0.0260572			
Ifitm7	0.6582268	0.0424333			
AK038388	0.6382835	0.0406771			
Pfkfb4	0.6374585	0.0406771			
Scarb1	0.6315394	0.0208386			
Tmem106a	0.6180528	0.0199841			
Cd300d*	0.6149989	0.0411697			
TC1486688	0.6117544	0.0260572			
Myh9	0.6091942	0.0314247			
Tia1	0.6088532	0.0354384			

Csf2rb1	0.6082428	0.0379543			
Pctk3	0.6079319	0.0251503			
AK089867	0.6061738	0.040137			
Pstk	0.6043853	0.0291848			
AK045243	0.5816074	0.0354384			
Cd244	0.5794334	0.0304426			
NAP045951-1	0.5753181	0.040137			
Irak3	0.5752363	0.0497032			
Comt*	0.5720299	0.0283388			
Lyl1	0.5654871	0.0387303			
D030029J20Rik	0.5613431	0.0424369			
NAP029356-1	0.5587797	0.0408644			
C130026L21Rik	0.5581975	0.0445632			
*Apob48r**	0.5575131	0.026206			
Fgl2	0.5385596	0.0465107			
B230208L21Rik	0.5305764	0.0424369			
Tmem37	0.5286151	0.0390745			
Ctsb	0.514308	0.0406771			
D8Ertd354e	0.5095144	0.0459494			
Gpr146	0.5041779	0.0342793			
Atp13a2	0.5021957	0.0334709			
Lyn	0.4847448	0.0289639			
Pars2	0.4803258	0.046229			
Rab3a	0.4800285	0.0464859			
Eif4ebp1	0.4763669	0.0424369			
Zfp3	0.4747451	0.0430313			
Tnfrsf19l	0.4703432	0.0283494			
Hectd2	0.4681382	0.0463652			
Ppt2	0.4584579	0.0360263			
Myo7a	0.4575087	0.0465408			
Cd151	0.4495158	0.0478532			
Slc16a10	0.4308468	0.0444629			
Pscd4	0.4060573	0.0490856			
Abcg1	0.3776486	0.0478532			

Supplementary Figure S4. Network analysis of significantly enriched gene sets after *per os* tBHQ treatment in splenic $CD4^+$ lymphocytes. The network contains 56 nodes. Nodes represent GO pathways significantly changed in gene set enrichment analysis ($p<0.05$, false discovery rate <0.25). Green nodes represent GO terms, grey edges connection between them. The size of the nodes is proportional with the number of genes in the GO term and the thickness of grey edges represents the number of common genes between two GO terms ($FDR=0.25$, $p<0.05$, edge cutoff 0.5).

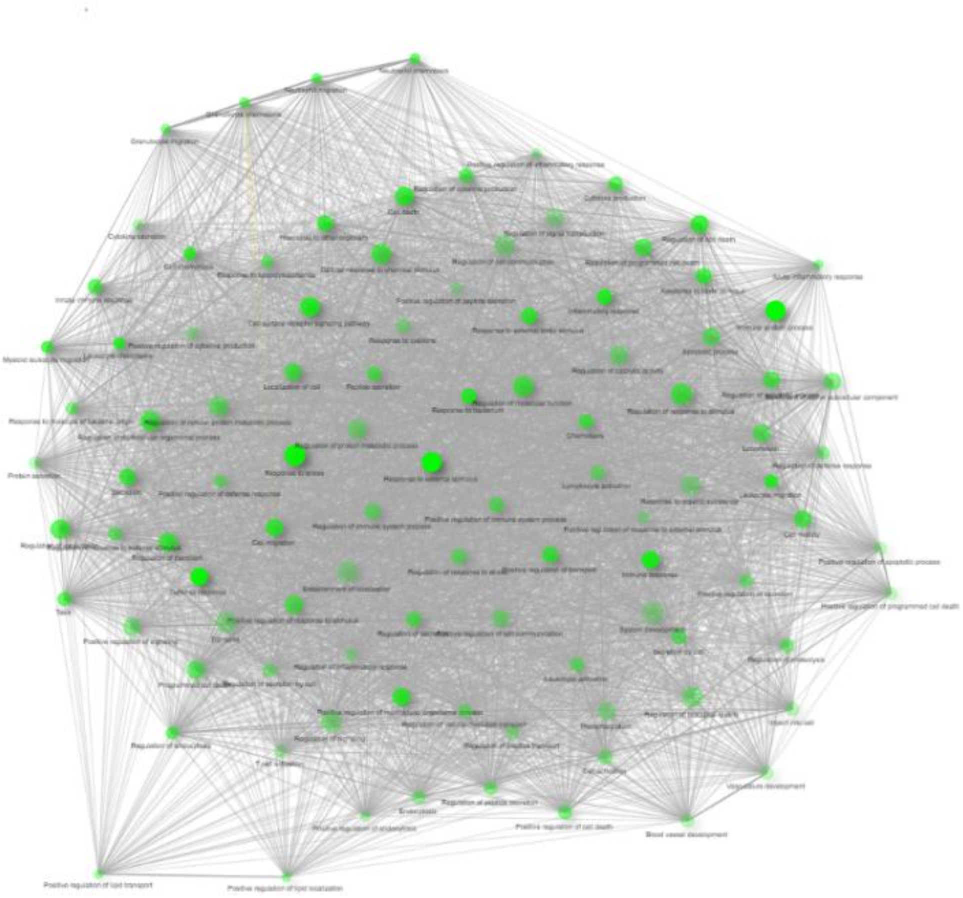

Supplementary Figure S5. Additional subnetworks from Network analysis. Nodes represent GO pathways significantly changed in gene set enrichment analysis (p<0.05, false discovery rate <0.25). Green nodes represent GO terms, grey edges connection between them. The size of the nodes is proportional with the number of genes in the GO term and the thickness of grey edges represents the number of common genes between two GO terms (FDR=0.25, p<0.05, edge cutoff 0.5).

A. Regulation of cell signaling

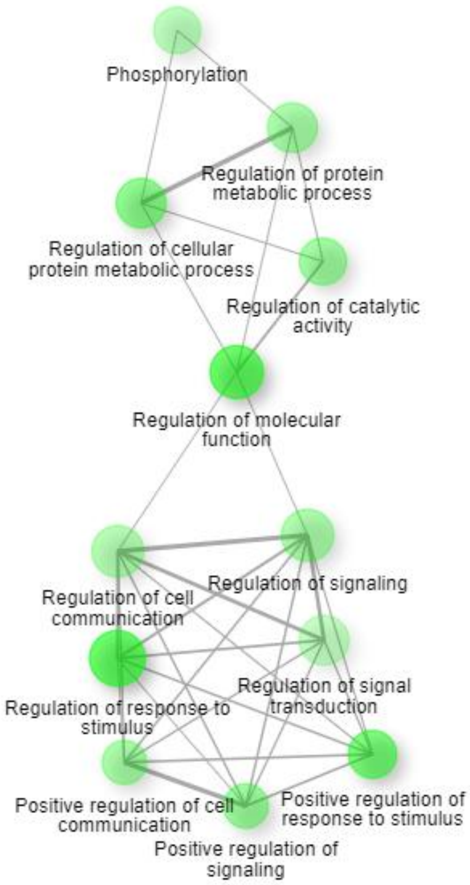

B. Secretion and transport

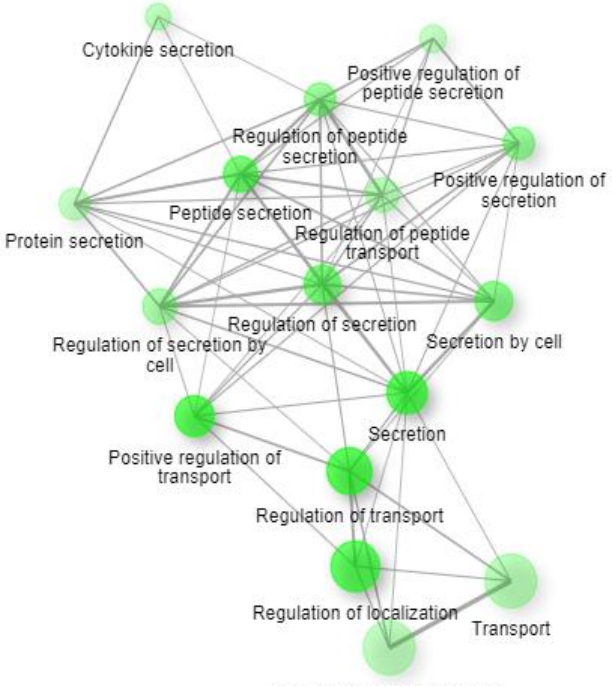

Supplementary Table S2. Significantly changed GO, KEGG, Reactome, TF, WP and CORUM terms after g:Profiler analysis [64]. Input genes were from Table 1 (significantly changed after tBHQ treatment and those that are related to influenza or SARS-CoV-2). A.) Influenza, B.) SARS-CoV-2.

A. Influenza

Source	Term_name	Adjusted_p_value
**GO:BP**	inflammatory response	5.64E-07
**GO:BP**	defense response	3.17285E-05
**GO:MF**	prostaglandin-endoperoxide synthase activity	0.000133097
**GO:BP**	innate immune response	0.001645605
**WP**	Photodynamic therapy-induced NF-kB survival signaling	0.002947529
**GO:BP**	immune system process	0.00327487
**GO:BP**	response to other organism	0.003615254
**GO:BP**	response to external biotic stimulus	0.003670657
**GO:BP**	response to biotic stimulus	0.00443811
**WP**	Fibrin complement receptor 3 signaling pathway	0.005907833
**WP**	Prostaglandin synthesis and regulation	0.00632312
**GO:BP**	biological process involved in interspecies interaction between organisms	0.007312913
**KEGG**	Transcriptional misregulation in cancer	0.007621782
**GO:BP**	defense response to other organism	0.007698772
**GO:BP**	cellular response to bacterial lipoprotein	0.010159674
**GO:BP**	response to bacterial lipopeptide	0.010159674
**GO:BP**	cellular response to bacterial lipopeptide	0.010159674
**GO:BP**	response to bacterium	0.010453143
**GO:MF**	peptidase activity	0.018413225
**GO:BP**	positive regulation of receptor binding	0.018949096
**GO:BP**	response to bacterial lipoprotein	0.018949096
**WP**	AGE/RAGE pathway	0.019942137
**GO:BP**	immune response	0.021481211
**KEGG**	NF-kappa B signaling pathway	0.023179192
**GO:BP**	cyclooxygenase pathway	0.024353083
**GO:BP**	regulation of cell migration	0.029835374
**GO:BP**	response to lipoteichoic acid	0.030428808
**GO:BP**	cellular response to lipoteichoic acid	0.030428808
**GO:BP**	regulation of localization	0.032330113
**GO:BP**	positive regulation of cell migration	0.032875638
**REAC**	Immune System	0.033089112
**GO:BP**	response to external stimulus	0.037708531
**GO:BP**	positive regulation of cell motility	0.040765128
**GO:BP**	cell migration	0.041761747

**GO:BP**	regulation of cell motility	0.0428563
**REAC**	Synthesis of Prostaglandins (PG) and Thromboxanes (TX)	0.043553629
**GO:BP**	positive regulation of cellular component movement	0.045615296
**GO:BP**	positive regulation of locomotion	0.046795933
**CORUM**	COX1 homodimer complex	0.049947455
**CORUM**	PTGS2 homodimer complex	0.049947455

B. SARS-CoV2

Source	Term_name	Adjusted_p_value
**GO:BP**	defense response	0.000865474
**GO:BP**	viral entry into host cell	0.001508618
**GO:BP**	entry into host	0.001816696
**GO:BP**	immune response	0.00205686
**GO:BP**	immune system process	0.002310072
**GO:BP**	movement in host environment	0.002392321
**GO:CC**	lytic vacuole	0.002971444
**GO:CC**	lysosome	0.002971444
**GO:BP**	biological process involved in interaction with host	0.003613031
**TF**	Factor: Erg; motif: NRRSAGGAAGNGG; match class: 1	0.004826403
**GO:CC**	vacuole	0.005442771
**GO:CC**	external side of plasma membrane	0.006186628
**GO:CC**	cell surface	0.008500716
**GO:BP**	biological process involved in interspecies interaction between organisms	0.013102578
**GO:CC**	endosome lumen	0.017800021
**GO:BP**	biological process involved in symbiotic interaction	0.019718536
**GO:BP**	defense response to other organism	0.022349117
**WP**	Network map of SARS-CoV-2 signaling pathway	0.026302756
**GO:BP**	viral life cycle	0.029748527
**GO:CC**	side of membrane	0.030316127
**GO:BP**	response to stress	0.035059475
**GO:BP**	response to external stimulus	0.035839933

Supplemmentary Table S3. Putative AHR and NRF2 binding sites on the genes listed in Table 1. After analysls with GeneHancer, those genes were selected from the list, that had 'elite' enhancer–gene relations reflecting both a high-likelihood enhancer definition and a strong enhancer–gene association. A Influenza, B SARS-CoV2

A. Influenza

	**AhR**			**Nrf2**		
**Gene symbol**	**GH Identifier**	**GH Type**	**GH Score**	**GH Identifier**	**GH Type**	**GH Score**
CtsB	GH08J011856	Promoter/Enhancer	2.1	GH08J011856	Promoter/Enhancer	2.1
	GH08J011851	Promoter/Enhancer	1.6	GH08J011876	Promoter/Enhancer	1.9
	H08J011843	Enhancer	1	-	-	-
Cd14	-	-	-	-	-	-
Cd151	GH11J000839	Promoter/Enhancer	2	GH11J000824	Promoter/Enhancer	1.9
	H11J001301	Promoter/Enhancer	2.1	-	-	-
	GH11J000794	Promoter/Enhancer	2	-	-	-
Cd209d	GH19J007521	Promoter/Enhancer	2.3	-	-	-
	GH20J060078	Enhancer	0.9	-	-	-
Ifitm1	GH11J000352,	Promoter/Enhancer	1.6	-	-	-
Ifitm2	GH11J000352	Promoter/Enhancer	1.6	-	-	-
Cxcr2 (Il8rb)	-	-	-	-	-	-
Osm	-	-	-	GH22J030193	Enhancer	1.5
	-	-	-	GH22J030201	Promoter/Enhancer	2.1
	-	-	-	GH22J030263	Enhancer	1
Padi4	-	-	-	GH01J017347	Enhancer	1
Perforin	-	-	-	-	-	-
Ppp1r3d	-	-	-	GH20J059938	Promoter/Enhancer	2.2
	-	-	-	GH20J060054	Promoter/Enhancer	1.6
Syn2	-	-	-	GH03J012191	Enhancer	1.3

B. SARS-CoV2

	Ahr			Nrf2		
**Gene symbol**	**GH Identifier**	**GH Type**	**GH Score**	**GH Identifier**	**GH Type**	**GH Score**
Anxa2	GH15J060388,	Promoter/Enhancer	2.1	GH15J060388	Promoter/Enhancer	2.1
	H15J060476	Promoter/Enhancer	1.9	GH15J060368	Promoter/Enhancer	1.5
	-	-	-	GH15J060525	Enhancer	1.2
Cd14	-	-	-	-		
Dusp10	-	-	-	GH01J221687	Enhancer	1.1
	-	-	-	H01J221986	Enhancer	1.4
Fpr-rs2	-	-	-	-	-	-
Gzmb	-	-	-	-	-	-
Mgl1	-	-	-	-	-	-
Mmp7	GH15J060388,	Promoter/Enhancer	2.1	-	-	-
Mmp9	H15J060476	Promoter/Enhancer	1.9	-	-	-
Otud	-	-		-	-	-
Ptgs1	GH09J122346	Enhancer	1.1	-	-	-
Ptgs2	-	-	-	-	-	-
Rel	H02J061535	Promoter/Enhancer	2.3	GH02J061535	Promoter/Enhancer	2.3
	H02J061015	Promoter/Enhancer	2			
Tirap	GH11J125624	Promoter/Enhancer	2	GH11J126350	Promoter/Enhancer	2
Trem1	-	-	-	-	-	-
Tnfsf13 (April)	H17J007571	Promoter/Enhancer	2	-	-	-
	GH17J007556	Promoter/Enhancer	2	-	-	-
	GH17J007347	Promoter/Enhancer	2.1	-	-	-
Usp38	-	-	-	GH04J143183	Promoter/Enhancer	1.8
